# Effect of the mitochondrial unfolded protein response on hypoxic death and mitochondrial protein aggregation

**DOI:** 10.1038/s41419-021-03979-z

**Published:** 2021-07-15

**Authors:** Junyi Yan, Chun-Ling Sun, Seokyung Shin, Marc Van Gilst, C. Michael Crowder

**Affiliations:** 1 Department of Anesthesiology and Pain Medicine, University of Washington School of Medicine, Seattle, WA 98109 USA; 2 Mitochondrial and Metabolism Center, University of Washington School of Medicine, Seattle, WA 98109 USA; 3 Department of Anesthesiology, Central Hospital of Changdian, 118214 Dandong, Liaoning China; 4 Department of Anesthesiology and Pain Medicine, Anesthesia and Pain Research Institute Yonsei University College of Medicine, 50-1 Yonsei-ro, Seodaemun-gu, Seoul, 03722 Korea; 5 Department of Genome Science, University of Washington School of Medicine, Seattle, WA 98109 USA

**Keywords:** Necroptosis, Energy metabolism

## Abstract

Mitochondria are the main oxygen consumers in cells and as such are the primary organelle affected by hypoxia. All hypoxia pathology presumably derives from the initial mitochondrial dysfunction. An early event in hypoxic pathology in *C. elegans* is disruption of mitochondrial proteostasis with induction of the mitochondrial unfolded protein response (UPR^mt^) and mitochondrial protein aggregation. Here in *C. elegans*, we screen through RNAis and mutants that confer either strong resistance to hypoxic cell death or strong induction of the UPR^mt^ to determine the relationship between hypoxic cell death, UPR^mt^ activation, and hypoxia-induced mitochondrial protein aggregation (HIMPA). We find that resistance to hypoxic cell death invariantly mitigated HIMPA. We also find that UPR^mt^ activation invariantly mitigated HIMPA. However, UPR^mt^ activation was neither necessary nor sufficient for resistance to hypoxic death and vice versa. We conclude that UPR^mt^ is not necessarily hypoxia protective against cell death but does protect from mitochondrial protein aggregation, one of the early hypoxic pathologies in *C. elegans*.

## Introduction

Hypoxia, when extreme, will kill aerobic cells. The pathways whereby cells go from healthy to dead when they encounter hypoxia are many and complex. By the time cells are dying from hypoxia, virtually every energy dependent process in the cell is dysfunctional. Pathological processes that occur early in hypoxic exposure when the pathology may still be reversible are of particular interest. This pathologic time course certainly varies by cell type and organism. In mammals, central nervous system neurons and cardiac myocytes are exquisitely sensitive to hypoxia, and during complete ischemia begin dying in only a few minutes after dissolved oxygen is depleted [1, 2]. On the other hand, peripheral neurons and skeletal myocytes can endure hours of a similar reduction of oxygen and fully recover. Some organisms can withstand prolonged severe hypoxia while others cannot. Certain hibernating fish and reptiles modulate anabolic and catabolic pathways and can tolerate near anoxic conditions over the winter months, while living in normal oxygen levels in the summer [3]. The primary cellular determinants of this enormous range in hypoxia tolerance are not well defined.

Mitochondria lie at the center of hypoxic biology and are logically the first organelle affected when oxygen levels fall below a crucial threshold. The vast majority of oxygen in metazoan cells is consumed by mitochondrial cytochrome C oxidase enabling the formation of ATP by ATP synthase. When oxygen levels fall, cytochrome C oxidase function fails, ATP levels decrease, and reactive oxygen species (ROS) are produced [4]. Besides ROS, the ATP deficit generated by hypoxia creates havoc throughout the cell including in the mitochondria where ATP-dependent processes such as the proton gradient, protein transport, synthesis, and folding are disrupted. The degree to which the failure of each of these processes contributes to hypoxic cell death is unclear.

In the nematode *C. elegans*, we and others have identified mutants and RNAis that protect worms from hypoxic cellular and organismal injury [5–25]. Not surprisingly, what is clear from these studies is that multiple pathways regulate hypoxic sensitivity. However, to what degree the pathological state of the mitochondria influences these pathways and vice versa has not been systematically examined. We have previously shown that hypoxia disrupts mitochondrial proteostasis [6, 26]. This disruption is evident by hypoxic induction of the mitochondrial unfolded protein response UPR^mt^ [26], a conserved adaptive pathway that responds to an increase in mitochondrial unfolded protein levels by increasing the synthesis of mitochondrial chaperones, proteases, and other proteins to restore proteostasis [27]. Hypoxia also promotes aggregation of mitochondrial proteins [6, 26]. Both UPR^mt^ activation and hypoxia-induced mitochondrial protein aggregation (HIMPA) occur well before there is evidence of cell death in *C. elegans* or permanent behavioral deficits [6, 12]. Thus, disruption of mitochondrial proteostasis occurs relatively early in the hypoxic pathological cascade, but the consequence of disruption of mitochondrial proteostasis and HIMPA on downstream cellular pathology, most importantly cell death is unclear. Even the relationship of the UPR^mt^ to HIMPA is not entirely clear. Activation of the UPR^mt^ by three RNAis targeting mitochondrial proteins reduced HIMPA and protected from hypoxic death [6]. However, loss of function of *atfs-1*, a gene which is thought to be essential for UPR^mt^ activation, also decreased HIMPA while *atfs-1* gain-of-function mutants increased HIMPA [6]. Thus, whether the UPR^mt^ promotes HIMPA or inhibits it is unclear with the relatively small number of UPR^mt^ activators examined. Here, we make use of the wide variety of *C. elegans* RNAis and mutants that have been shown to be hypoxia protective, or to induce the UPR^mt^ to address three fundamental questions about the relationship between mitochondrial proteostasis and hypoxic injury. Is activation of the UPR^mt^ a necessary component of hypoxia protective mechanisms? Is UPR^mt^ activation itself hypoxia protective? Does the UPR^mt^ promote or reduce HIMPA?

## Results

### UPR^mt^ activation not necessary for hypoxia resistance

**Fig. 1:**
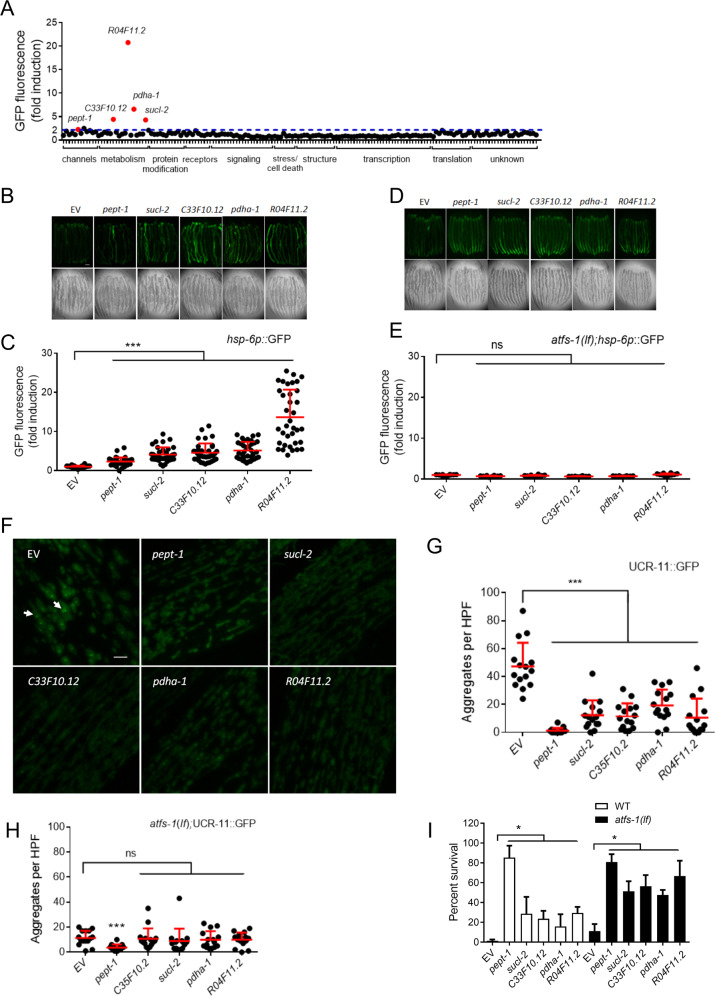
Only a small subset of hypoxia resistant RNAis activate the UPR^mt^. **A** Fluorescence screen of RNAis for UPR^mt^ activation. *hsp-6p*::GFP induction was quantified for 161 Hyp RNAi clones with the functional categories as originally assigned [9]. Fold induction (mean) of GFP fluorescence is relative to empty vector (EV) RNAi (L4440) (*n* = 10). Expression of GFP over 2-fold (blue dash line) of empty vector is considered to be positive. Five positive hits confirmed in 2nd screen are shown in red. **B** Examples of *hsp-6p*::GFP expression induced by knockdown of the five positive RNAi clones. *zcIs13*[*hsp-6p::*GFP] worms were placed onto RNAi bacteria from egg and imaged as young adults 3 days later. Bar, 0.1 mm. **C** Quantification of GFP expression by corresponding RNAis as in **B**. Data (mean ± SD) were obtained from four independent experiments, *n* = 30 animals; ***p < 0.001 with pooled data shown and *p*-values were calculated using unpaired *t*-test. **D** Examples of *zcIs13*[*hsp-6p::*GFP] GFP expression induced by knockdown of positive five RNAi clones in *atfs-1*(*tm4919*) background. Worms were placed onto RNAi bacteria from egg and imaged 3 days later. **E** Quantification of GFP expression by corresponding RNAis in *atfs-1*(*tm4919*) background. Data (mean ± SD) were obtained from five independent experiments, *n* = 30 animals. **F** Examples of HIMPA in body wall muscles after hypoxia of indicated RNAi treated *gcIs46* worms. Green, UCR-11::GFP. Arrow, UCR-11::GFP aggregate. Bar, 2 μm. **G** Quantification of aggregate numbers as in **F**. *n* = 15 animals from three independent experiments; ***p < 0.001. with pooled data shown. *P*-values were calculated using unpaired *t*-test. **H** Quantification of HIMPA by UCR-11::GFP after hypoxia in body wall muscles of indicated RNAi treated *atfs-1(tm4919)*; *gcIs46* worms. *n* = 15 animals from three independent experiments; ***p < 0.001. with pooled data shown. *P*-values were calculated using unpaired *t*-test. **I** Hypoxia resistance is maintained in *atfs-1*(lf) background. Wild-type (N2) or *atfs-1*(*tm4919*) treated with indicated RNAis and hypoxic survival was scored. *n* = 9 plates of worms (>100 worms/plate) from three independent trials. *p < 0.05, unpaired *t*-test.

We have previously performed a forward genome-wide screen in *Caenorhabditis elegans* for genes, whose inactivation by RNAi conferred a hypoxia resistance phenotype (HypR) and identified about 200 such genes [9]. To explore the hypothesis that activation of the UPR^mt^ might be a common or even invariant component of the mechanism underlying hypoxia resistance of these RNAis, we screened through all the HypR RNAis that we could recover from the library and confirm their identity by sequence. Hundred and sixty-one of the HypR RNAi were confirmed and tested for activation of the mitoUPR under normoxic conditions as assessed by the fluorescence levels of the mitoUPR transcriptional reporter *hsp-6*p::GFP (*hsp-6* is the ortholog of human mitochondrial chaperone HSP70 and is a canonical component of the UPR^mt^) [28–31]. RNAi knockdown of only five genes reproducibly induced the mitoUPR reporter (Fig. 1A). Thus, mitoUPR activation, at least as assessed by the *hsp-6* reporter, is by no means necessary for hypoxia resistance.

The five RNAis that induced the mitoUPR were against *pept-1, sucl-2, C33F10.12, pdha-1, and R04F11.2* (Fig. 1A and Supplementary Table S1). *pept-1* encodes an oligopeptide transmembrane transporter, localized to the apical plasma membrane in the intestine [32]. Reduction of function of *pept-1* has been found to reduce protein synthesis rates and levels of essential amino acids [5, 33]. A *pept-1* loss of function mutant was recently isolated in a screen for hypoxia resistant mutant worms, and its essential role in normal rates of protein synthesis was confirmed [5]. How loss of function of *pept-1* activates the UPR^mt^ is unclear although recently the UPR^mt^ has been shown to regulate mitochondrial protein synthesis that would rely on amino acids, whose transport depends on *pept-1*. On the other hand, *sucl-2, C33F10.12, pdha-1, R04F11.2* all encode mitochondrial proteins that form or interact with multi-subunit proteins, where perturbations in stoichiometry have been shown to induce the UPR^mt^ [28, 30, 34]. Inactivation of these five genes by RNAi led to a range of UPR^mt^ reporter induction (Fig. 1B, C), from 2.3 to 13.7-fold that had no correlation with the previously reported levels of hypoxia resistance where *pept-1*(RNAi) was most resistant and *sucl-2*(RNAi) least resistant to hypoxia [9]. *atfs-1* is a transcription factor required for UPR^mt^ activation [35, 36]. The induction of *hsp-6p*::GFP by the five UPR^mt^-activating HypR RNAis was fully suppressed by an *atfs-1* loss-of-function mutation (Fig. 1D, E), indicating that the induction of *hsp-6p*::GFP by the five Hyp RNAis is acting through the canonical UPR^mt^ pathway.

Hypoxia induces mitochondrial protein aggregation (HIMPA) [6, 26]. We found that inactivation of all five UPR^mt^-activating HypR genes strongly lowered HIMPA as assessed by the *ucr-11*::GFP mitochondrial protein aggregation reporter [6] (Fig. 1F, G). As shown previously, *atfs-1*(lf) also reduced the aggregate levels; only *pept-1*(RNAi) significantly reduced aggregates further in the *atfs-1*(lf) background indicating that at least part of the effect of *pept-1*(RNAi) is independent of *atfs-1* (Fig. 1H). *atfs-1*(lf) did not suppress the hypoxia resistance of any of the five RNAis; thus, neither *atfs-1* function nor UPR^mt^ activation is essential to their mechanisms of hypoxia resistance (Fig. 1I). These results suggest that while resistance to hypoxic cell death and HIMPA may be mechanistically associated and that a functional UPR^mt^ may be critical for aggregate formation, the UPR^mt^ is not required for protection from hypoxia and in general activation of the UPR^mt^ does not appear to commonly accompany a hypoxia-resistant phenotype in *C. elegans*.

### UPR^mt^ activation reduces HIMPA

**Fig. 2:**
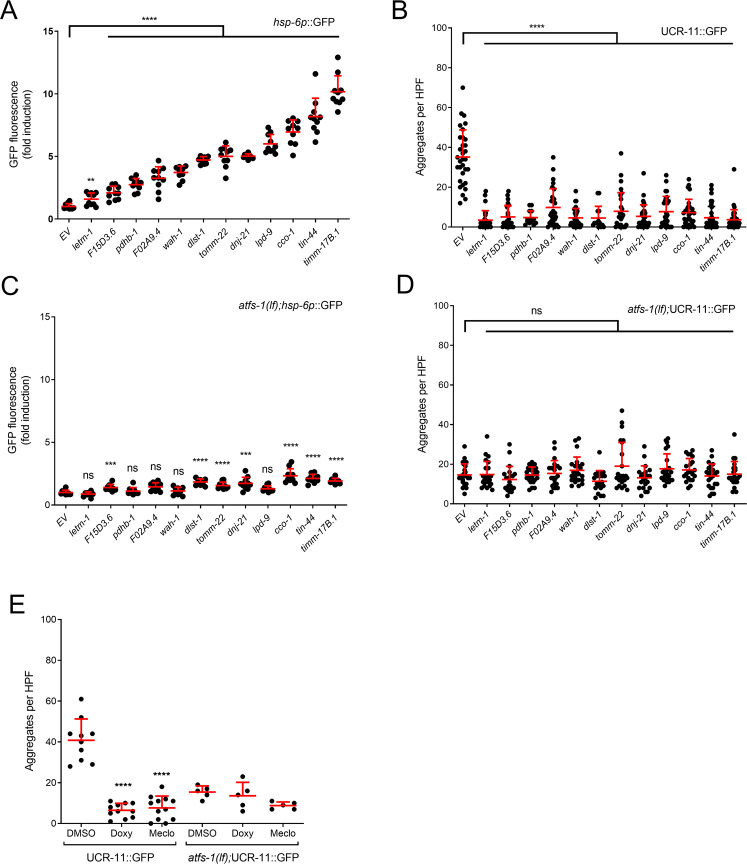
UPR^mt^ activation Reduces HIMPA. **A** UPR^mt^-activating RNAis as assessed by *hsp-6p*::GFP expression. *hsp-6p*::GFP induction was quantified for 12 RNAis previously shown to induce the UPR^mt^. Fold induction of GFP fluorescence is the mean fluorescence relative to empty vector RNAi (L4440) (*n* = 10). Data were obtained from 10–36 individual animals for all panels; mean ± SD; **p < 0.01, ****p < 0.0001, unpaired *t*-test. **B** UCR-11:GFP HIMPA reduced by the UPR^mt^ activating RNAis. **C** UPR^mt^ activation is partially or completely blocked in the *atfs-1(tm4919* lf*)* animals. **D** *atfs-1(*lf*)* reduces aggregates and suppresses a further reduction in aggregates by UPR^mt^-activating RNAis. **E** Mitochondrial translation inhibitors (doxycycline and meclocycline) reduce HIMPA in a non-additive manner along with *atfs-1*(lf). **p < 0.01, ***p < 0.001, ****p < 0.0001, n or ns not significant versus EV @ Bonferroni-corrected *p* < 0.01, unpaired *t*-test.

To examine more broadly the role of the UPR^mt^ in HIMPA, we tested 12 RNAis previously shown to activate the UPR^mt^ (Supplementary Table S2) [37]. We first confirmed that all 12 RNAis did indeed activate the UPR^mt^ as measured by *atfs-1*-dependent induction of the *hsp-6*::GFP transcriptional reporter (Fig. 2A, C). All 12 UPR^mt^-activating RNAis strongly reduced HIMPA (Fig. 2B). Unlike for the *pept-1*(RNAi), none of the 12 RNAis significantly reduced HIMPA further in the *atfs-1*(*lf*) background (Fig. 2D). Again, *atfs-1*(lf) paradoxically reduced HIMPA in the no RNAi empty vector condition but not to the same degree of reduction as the UPR^mt^ RNAis. The mitochondrial translation inhibitors doxycycline and meclocycline induce the UPR^mt^ in an *atfs-1*-dependent manner (Supplementary Fig. S1) [26, 38]. Likewise, doxycycline and meclocycline reduced HIMPA and did not further reduce the aggregate levels in *atfs-1*(lf) animals (Fig. 2E). Thus both genetic and pharmacological UPR^mt^ activators reduce HIMPA, while at the same time the UPR^mt^ master regulator gene *atfs-1* paradoxically promotes HIMPA, as previously reported [6].

### Activation of UPR^mt^ does not necessarily confer hypoxia resistance

**Fig. 3:**
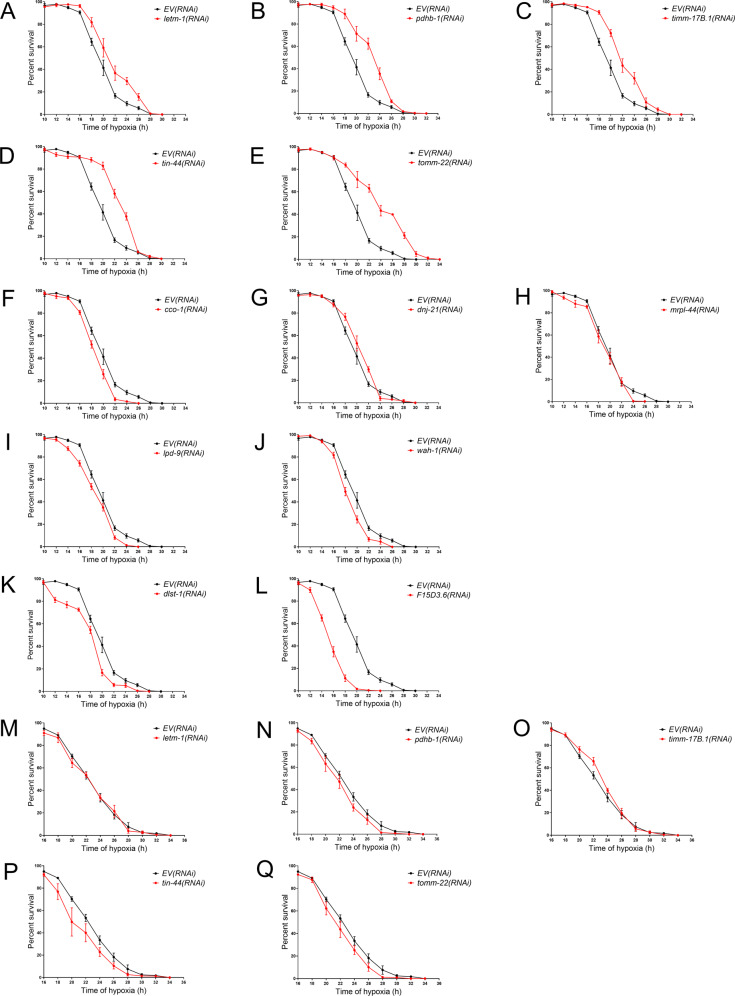
Activation of UPR^mt^ does Not Necessarily Produce Hypoxia Resistance. The twelve UPR^mt^-activating RNAis are tested for hypoxic sensitivity in the wild type background **A–L** and in the *atfs-1*(lf) mutant background **M–Q**; significance @ *p* < 0.0001, F-test, simultaneous nonlinear regression; data for each point is from 3 to 12 independent trials with at least 100 animals/trial, mean +/− sem. **A**–**E** Five RNAis produced significant hypoxia resistance; **F**–**J** Five RNAis have no significant effect on hypoxic sensitivity; and **K**, **L** Two RNAis produced significant hypersensitivity to hypoxia. **M–Q** The hypoxia resistance of the five RNAis is suppressed by *atfs-1*(lf).

We tested whether the 12 UPR^mt^-activating RNAis were protective from hypoxic organismal death like the five UPR^mt^-activating HypR RNAis and like doxycycline and meclocycline [9, 26, 38]. Five RNAis (*letm-1, pdhb-1, timm-17B1, tin-44*, and *tomm-22*) produced significant hypoxia resistance (Fig. 3A–E); five RNAis (*cco-1, dnj-21, mrpl-44, lpd-9*, and *wah-1*) showed no significant effect on hypoxic sensitivity (Fig. 3F–J); 2 RNAis (*dlst-1, F15D3.6*) produced hypersensitivity to hypoxia (Fig. 3K, L). Interestingly, unlike for the UPR^mt^-activating RNAis identified in our screen for strongly hypoxia resistant RNAis (Fig. 1), the relatively weak hypoxia resistance of these UPR^mt^-activating RNAis was *atfs-1*-dependent (Fig. 3M–Q). Thus, our data shows that activation of the UPR^mt^ may in some genetic contexts confer *atfs-1*-dependent mild hypoxia protection, but this is by no means a necessary consequence of UPR^mt^ activation.

### TMRE stains mitochondrial aggregates

**Fig. 4:**
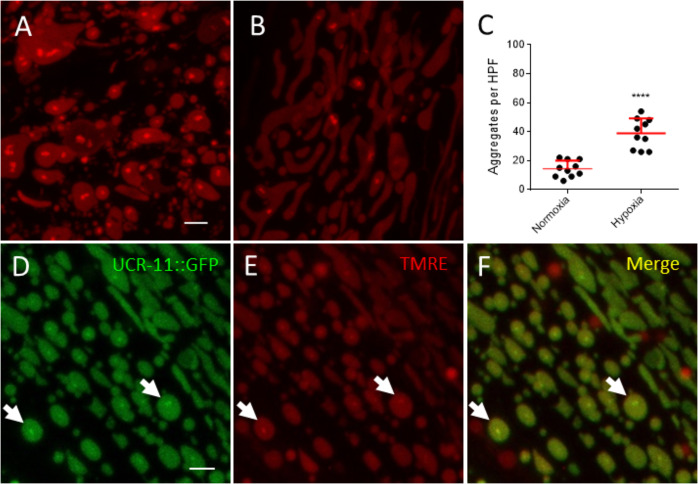
TMRE Stains HIMPA. (Scale bar). **A** Wild type worms exposed to 12 h of hypoxia, stained with TMRE and mounted immediately. TMRE stains the mitochondria and HIMPA. **B** Normoxic wild type worms (not exposed to hypoxia) stained with TMRE and mounted immediately. Few TMRE-stained aggregates in normoxic wild type animals. **C** Quantitation of aggregates in wild type worms. Mean ± SD of ten animals ****p < 0.0001, unpaired *t*-test. **D** HIMPA visible by GFP in *gcIs46*[UCR-11::GFP]. **E** HIMPA visible with TMRE staining in *gcIs46*. **F** Merge of **D** and **E**. 79.7% of UCR-11::GFP aggregates colocalized with TMRE aggregates in muscle (*n* = 1472). 93.5% of TMRE aggregates in muscle colocalized with UCR-11::GFP aggregates (*n* = 1255). Number of images = 50. Scale bars = 2 µm.

While examining the effect of hypoxia on mitochondrial membrane potential, we discovered that the voltage sensitive mitochondrial dye tetramethylrhodamine ethyl ester (TMRE) not only stained functional mitochondria, but also stained hypoxia-induced mitochondrial protein aggregates (Fig. 4A–C). This finding in the wild type N2 strain importantly demonstrates that aggregates occur in an animal with native mitochondrial proteins. We then compared the aggregates visualized with TMRE to those visible with the *ucr-11*::GFP reporter. The TMRE-stained aggregates strongly colocalized with UCR-11::GFP aggregates (Fig. 4D–F). Besides demonstrating importantly that the UCR-11::GFP aggregates are not an artifact of an overexpressed and GFP-tagged protein, TMRE staining can be used in all *C. elegans* strains to detect mitochondrial aggregates and potentially in other models of hypoxic injury.

### HIMPA blocked by hypoxia resistance without UPR^mt^ activation

**Fig. 5:**
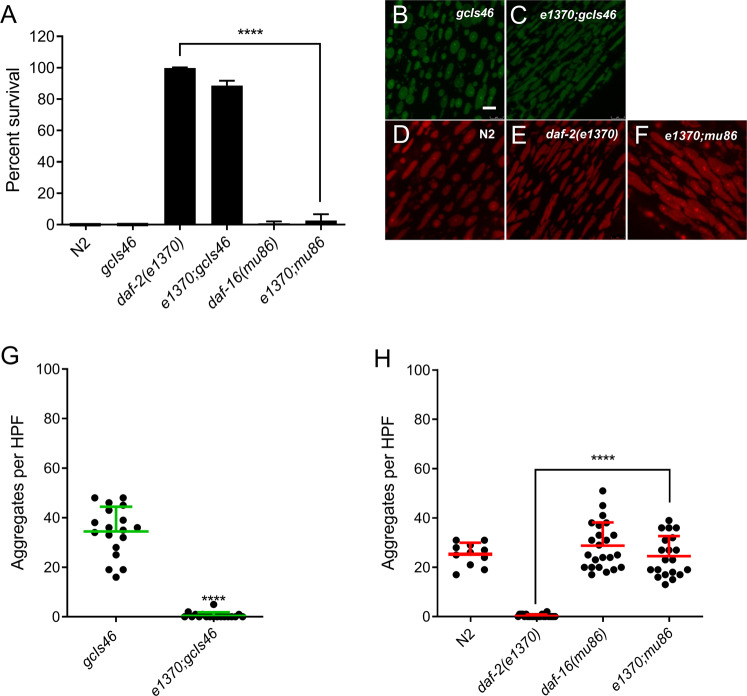
Reduction-of *daf-2* function Reduces HIMPA. **A** As previously reported [12], *daf-2(e1370)* animals are hypoxia resistant in a *daf-16* dependent manner and in the *gcIs46* background (mean ± SD, *n* = 4–12 independent trials). **B**, **C** Comparison of *gcIs46*::GFP HIMPA in the wild type **B** and *daf-2(e1370)* background **C**. **D**–**F** Comparison of HIMPA by TMRE in wild type (N2), *daf-2(e1370)*, and in *daf-2(e1370);daf-16(mu86)*. **G** Quantitation of GFP HIMPA in *gcIs46* and *daf-2(e1370);gcIs46* (mean ± SD of 18 animals). **H** Quantitation of TMRE HIMPA in the strains with the noted genotypes (mean ± SD of 11–24 animals). ****p < 0.0001, unpaired *t*-test. Scale bars = 2 µm.

We have previously identified two distinct mechanisms, the insulin/IGF-receptor pathway and the translation machinery, that regulate cytoplasmic proteostasis and profoundly modulate hypoxic survival. The insulin/IGF-receptor pathway has been shown to strongly regulate hypoxic sensitivity, aging, and proteostasis in *C. elegans* [12, 23, 39, 40]*. daf-2* encodes the *C. elegans* insulin/IGF receptor, and *daf-2* reduction-of-function mutants are highly resistant to hypoxic injury via a mechanism that requires the FOXO transcription factor DAF-16 [12, 23]. To test whether the *daf-2* pathway affected HIMPA, we built the *daf-2(e1370*lf*)*;*gcIs46*[*ucr-11*p::GFP] strain, confirmed that it was strongly hypoxia resistant (Fig. 5A), and then scored for HIMPA. *daf-2(e1370)* strongly reduced HIMPA as measured by UCR-11::GFP and TMRE (Fig. 5B–E, G, H). HIMPA and hypoxic sensitivity were restored in the *daf-2(e1370);daf-16(mu86)* double mutant strain (Fig. 5A, F, H). *daf-2* RNAi, which is strongly protective from hypoxic injury [10], did not activate the UPR^mt^ and therefore the effect of *daf-2*(rf) on reducing aggregates is not explained by UPR^mt^ activation (Supplementary Fig. S2).

**Fig. 6:**
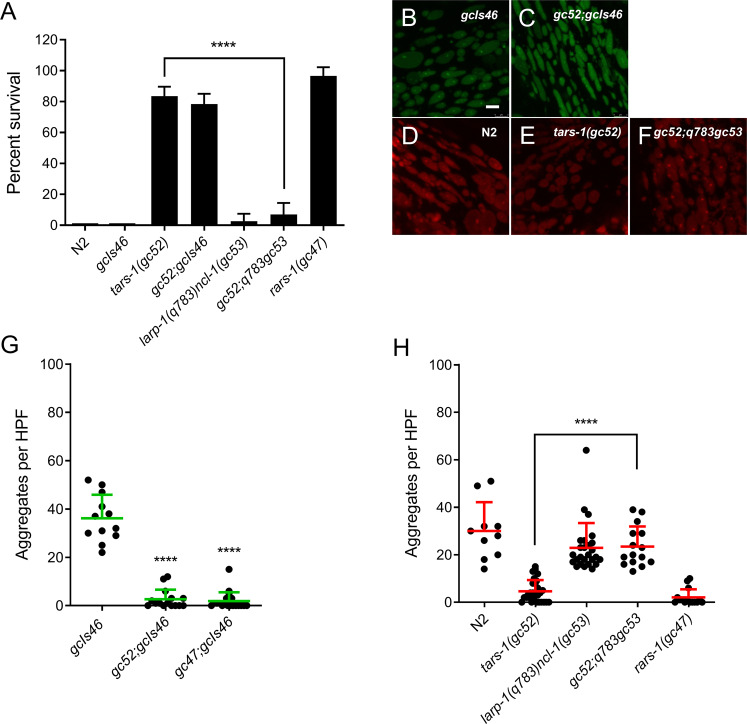
Loss-of function mutations in the translation machinery reduces HIMPA. **A** As previously reported [5, 11], *rars-1 (gc47)* and *tars-1(gc52)* animals are resistant to hypoxia and the resistance of *tars-1*(*gc52)* is maintained in a *gcIs46* background and is suppressed by *larp-1(q783)ncl-1(gc53)* (mean ± SD of 6–12 independent trial with >100 animals/trial). **B**, **C** Comparison of *gcIs46*::GFP HIMPA in the wild type (**B**) and *tars-1*(*gc52)* backgrounds (**C**). **D**–**F** Comparison of HIMPA by TMRE in wild type (N2), *tars-1*(*gc52)*, and in tars-1(gc52); *larp-1(q783)ncl-1(gc53)* animals **G** Quantification of GFP aggregates in *gcIs46* and *tars-1(gc52);gcIs46* (mean ± SD of 12–19 animals). **H** Quantification of TMRE aggregates in the strains with the noted genotypes (mean ± SD of 10–28 animals).****p < 0.0001, unpaired *t*-test. Scale bars = 2 µm.

The translation machinery has been repeatedly implicated in *C. elegans* hypoxic sensitivity [5, 8, 9, 11]. Translation machinery mutations and RNAis that reduce protein synthesis are strongly hypoxia protective [5, 11]. *tars-1(gc52)* and *rars-1(gc47)* carry reduction of function mutations in threonyl-aminoacyl and arginyl-aminoacyl tRNA synthetases, respectively, and are strongly resistant to hypoxic death (Fig. 6A), as previously shown [5, 11]. Both translation machinery mutants fully blocked HIMPA as measured by both UCR-11::GFP and TMRE (Fig. 6B–E, G, H). A combination of loss-of-function mutations in two negative regulators of the translation machinery, *larp-1* and *ncl-1* [5], suppresses the hypoxia resistance of *tars-1(gc52)* (Fig. 6A) as previously reported [5] and likewise restores HIMPA (Fig. 6F, H). As was seen for *daf-2*, RNAi knockdown of *rars-1* did not activate UPR^mt^ (Supplementary Fig. S2), demonstrating the effect on *rars-1* reduction-of-function on hypoxic death and HIMPA was not due to an activation of the UPR^mt^. These results indicate that hypoxia protective mechanisms of *daf-2/daf-16* signaling and reduction of translation do not require UPR^mt^ activation yet protect from HIMPA.

## Discussion

Our study shows that a hypoxia resistant phenotype in *C. elegans* is not commonly accompanied by UPR^mt^ activation and that UPR^mt^ activation is not always hypoxia protective. In other words, UPR^mt^ activation is neither necessary nor sufficient for protection from hypoxic cell death in *C. elegans*. However, among RNAis selected solely for their ability to induce the UPR^mt^ we did find a strong enrichment for hypoxia resistance, where five of the twelve UPR^mt^-activating RNAis conferred hypoxia resistance. By comparison, in a screen of over 16,000 RNAis, only 199 were found to reproducibly produce hypoxia resistance [9]. While these twelve are a small sample and the whole genome RNAi screen deliberately ignored weak hypoxia resistance phenotypes, this approximately 30-fold enrichment for hypoxia resistance suggests that UPR^mt^ activation may provide hypoxia protection in certain genetic backgrounds.

We also examined the effect of hypoxia resistance and UPR^mt^ activation on HIMPA. We found that resistance to hypoxic death and resistance to HIMPA were invariantly associated. All RNAis or mutants that were resistant to hypoxic death reduced HIMPA. Importantly, the strongly hypoxia resistant *daf-2, tars-1*, and *rars-1* mutants or RNAis block HIMPA without activating the UPR^mt^. Thus, UPR^mt^ activation is neither necessary for resistance to hypoxia nor for abrogation of HIMPA. We also found that all UPR^mt^-activating RNAis as well as doxycycline and meclocycline, which strongly activate the UPR^mt^, brought hypoxia-induced mitochondrial aggregates to near background levels. To be clear, both hypoxia-resistant and non-hypoxia resistant UPR^mt^-activating RNAis strongly reduced HIMPA. The effect of the RNAis on activation of the UPR^mt^ was mostly if not completely dependent on a functional *atfs-1* gene. This was to be expected as *hsp-6* activation is dependent on ATFS-1 [34, 36].

**Fig. 7:**
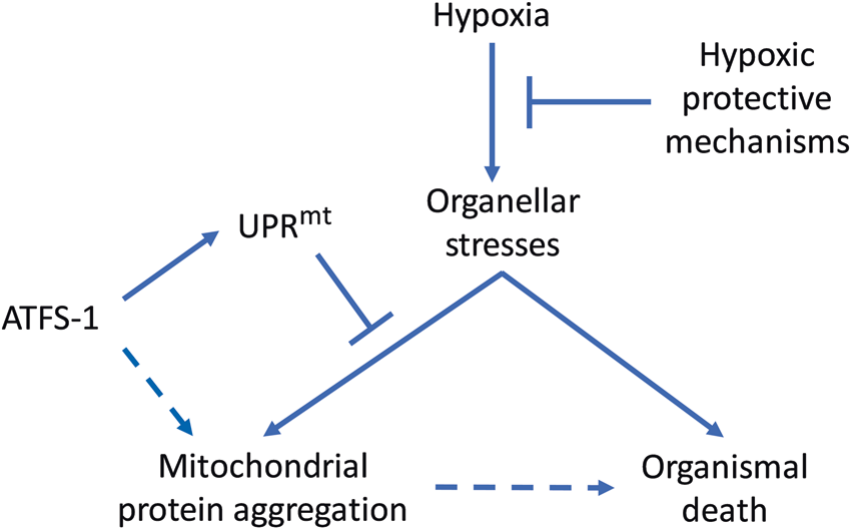
Hypoxia induces organellar stress that can manifest in the mitochondria as protein aggregation (HIMPA). ATFS-1, the UPR^mt^ master regulator, both reduces HIMPA by activating the UPR^mt^ and promotes HIMPA by an unknown mechanism. HIMPA may contribute to but is not required for cellular and organellar death.

The reduction in the aggregates by both UPR^mt^ activation and *atfs-1* loss-of-function is a puzzling paradox, albeit consistent with our previously published results [6]. We previously showed that *atfs-1* RNAi and the *atfs-1(tm4919)* loss-of-function mutant reduced HIMPA and an *atfs-1* gain-of-function mutant increased aggregates [6]. We had also previously shown that *cco-1, letm-1*, and F02A9.4 RNAis reduced HIMPA, but we attributed this effect to their hypoxia resistance or reduction in the levels of their corresponding proteins that might form the aggregates themselves [6]. We found here that of these three only *letm-1* RNAi reproducibly conferred hypoxia resistance yet all three RNAis, indeed all of the UPR^mt^ activating RNAis, strongly reduced HIMPA. Thus, clearly hypoxia resistance is not necessary for this effect. To reconcile that both *atfs-1*(lf) and *atfs-1*-dependent UPR^mt^ activation reduce aggregates, we hypothesize that ATFS-1 functions both to inhibit HIMPA by activating the UPR^mt^ and to promote HIMPA through some other unknown mechanism (Fig. 7). ATFS-1 could both function to reduce aggregates by activation of its downstream UPRmt targets, and in the absence of UPRmt activation function to promote HIMPA through an unknown mechanism. The fact that the level of activation of the *hsp-6*::GFP transcriptional reporter does not correlate with the ability to suppress hypoxia-induced aggregates (Fig. 2A, B) suggests that the HSP-6 chaperone levels are not directly responsible for the effect of the UPR^mt^ on aggregation, or that a very low threshold of *hsp-6* induction suppresses aggregation fully. Which *atfs-1*-regulated protein(s) might be responsible for promoting HIMPA is unknown; ATFS-1 has been shown to control the synthesis of a large number of proteins involved in mitochondrial proteostasis and mitochondrial function as well as some non-mitochondrial proteins [34, 41, 42]. Likewise, cytoplasmic and ER proteostasis pathways regulate the UPR^mt^ and mitochondrial proteostasis [42]. Thus, ATFS-1 may function in concert with one of those non-UPR^mt^ pathways to regulate HIMPA directly.

An important technical advance of this work is the discovery that TMRE stains HIMPA. TMRE is a lipophilic cationic dye that accumulates in the matrix of viable mitochondrial, where the membrane potential is highly negative relative to the intermembrane space [43, 44]. Besides membrane potential driven accumulation, TMRE has been shown to bind to both surfaces of the inner membrane; however, whether it is binding to lipids, proteins, or both at the inner mitochondrial membrane is unclear [43]. The aggregates appear to be in the matrix of the mitochondria although the resolution is inadequate to be certain. Other than UCR-11::GFP which strongly colocalizes with the TMRE-stained aggregates, we have no empirical evidence for what composes the TMRE-aggregates. In principle, TMRE can bind non-specifically to hydrophobic and negatively charged surfaces. Thus, denatured mitochondrial proteins, lipids, and even nucleic acids are reasonable candidates. Regardless of the nature of the binding target, the discovery that TMRE binds to hypoxia-induced mitochondrial aggregates rules out that HIMPA is an artifact of the GFP reporter overexpression. TMRE can be used to examine for HIMPA in any hypoxic/ischemic model without the need for expression of a tagged protein, electron microscopy, or immunofluorescence.

In summary, resistance to hypoxic organismal death and resistance to hypoxia-induced mitochondrial protein aggregation are strongly correlated. This suggests that HIMPA may be an early hypoxic pathology along the pathway to eventual hypoxic cell death. However, it is important to emphasize that we have not directly measured any aspect of mitochondrial function to determine whether HIMPA correlates with abnormalities in mitochondrial function. On the other hand, activation of the UPR^mt^ is neither necessary nor sufficient for hypoxia resistance but may weakly promote hypoxic survival in certain contexts. Activation of the UPR^mt^ did invariably reduce HIMPA whether or not it conferred resistance to hypoxic death. This effect of the UPR^mt^ favors a model, where the UPR^mt^ directly abrogates mitochondrial protein aggregation during hypoxia. Future investigation will continue to define the breadth of mechanisms that protect cells from hypoxic death, to define the components of the UPR^mt^ that inhibit HIMPA, and the ATFS-1-regulated mechanism that promotes it.

## Materials and methods

### *C. elegans* strains and culture methods

*C. elegans* strains were cultured and maintained at 20 °C on NGM agar with OP50 *E. coli* food unless otherwise noted [45]. The N2 (Bristol) strain was the standard wild-type strain from the Caenorhabditis Genetics Center (CGC, University of Minnesota). The mitochondrial UPR transcriptional reporter strain SJ4100 (*zcIs13*[*hsp-6p*::GFP]) was obtained from the CGC. *gcIs46* [*ucr-11*::GFP] and *atfs-1(tm4919*);*gcIs46* were generated as previously described [6]. *atfs-1*(*tm4919*);*zcIs13*, *daf-2(e1370)*;*gcIs46*, *rars-1(gc47);gcIs46*, and *tars-1(gc53);gcIs46* were generated in this study by routine genetic crosses and confirmation by phenotypes and/or PCR genotyping.

### Hypoxic incubations and hypoxic death

Synchronized young adult worms were subjected to hypoxia as described previously except that hypoxic incubation temperature was 26–26.5 °C [12, 46]. Briefly, each plate of worms was washed into a 1.5 ml tube with 1 ml of M9 buffer (22 mM KH_2_PO_4_, 22 mM Na_2_HPO_4_, 85 mM NaCl, and 1 mM MgSO_4_), and after the worms settled by gravity, all but 100 μl of M9 was removed. The tubes were then placed in the anaerobic chamber (Forma Scientific, Waltham, MA, USA) for incubation times ranging from 10 to 34 h as indicated. Oxygen tension was always ≤ 0.3%. Following the hypoxic incubation unless otherwise noted, worms were transferred using glass Pasteur pipettes onto seeded NGM plates and recovered at 20 °C for 24 h. Normoxic incubations were otherwise identical except performed in a 26.5 °C room air incubator. Organismal death was scored as previously described [46]. Briefly, animals were scored as dead if pharyngeal pumping, spontaneous and evoked movement (touching with a platinum wire) were not observed. For the mitochondrial aggregates assay, synchronized worms were kept on NGM plates and placed into anaerobic chamber for 12 h and then mounted for confocal imaging on glass slides [6].

### RNAi experiments

The bacterial RNAi feeding strain L4440 carrying the empty RNAi vector strain was from the Ahringer *C. elegans* RNAi library (MRC Gene service, Swindon, UK) and was used as the negative control (Empty Vector-EV) for all RNAi experiments [47]. Bacterial strains containing RNAi plasmids were cultured and induced with 0.1% β-lactose in 100 mg/ml ampicillin for 24 h at 23 °C as described previously [9]. Worms were synchronized on RNAi plates for 3.5 days (N2) or 4.5 days (*rars-1(gc47)* background) until reaching adulthood; worms not reaching adulthood were excluded.

### Mitochondrial UPR reporter and screening

SJ4100 (*zcIs13*[*hsp-6p*::GFP]) was used as a reporter for mitochondrial UPR activation [28]. Animals were synchronized from eggs by bleaching gravid adults, allowed to develop to adulthood, and then mounted on agar pads for imaging. All UPR^mt^ fluorescent reporter determinations were performed with worms exposed only to normoxic conditions. Briefly, a minimum of ten worms were mounted onto an agar pad on a standard microscope slide and imaged on a Zeiss axioskope 2 at ×10 magnification. Using ImageJ software (https://imagej.nih.gov/ij/), each worm was outlined and the fluorescence intensity within the area was measured and background intensity was then subtracted to obtain the total intensity/worm as previously described [26].

#### Chemical treatment and TMRE staining

Concentrated stocks of doxycycline (225 mM) and meclocycline (294 mM) in DMSO (Dimethyl sulfoxide) (Sigma-Aldrich Corp, St. Louis, MO, USA), were diluted to a final concentration of 100 μM with nuclease-free, deionized water and 300 µl was spread evenly onto a NGM plate seeded with OP50 onto which were placed synchronized eggs allowed to develop into 1-day-old adults. Plates containing TMRE (ThermoFisher, Waltham, MA, USA) were made the same way as described above with 300 µl at a final concentration of 5 μM spread onto the plate. For TMRE staining, 1-day-old synchronized worms were transferred onto the TMRE-containing plates for 2 h before the plates were placed into the anaerobic chamber for 12 h for hypoxic exposure.

### Aggregate imaging and counting

Images of UCR-11::GFP and TMRE staining were acquired with confocal microscopy as previously described [6, 26]. Briefly, paralysis was produced by mounting worms in a solution of 50 mM levamisole (Sigma-Aldrich Corp., St. Louis, MO, USA) in M9 prior to imaging. Images were acquired from at least 5–10 randomly selected worms at 1024 × 1024 resolution using a 63× objective with 8 × zoom producing a 23.07 × 23.07 μm image (defined as one high power field, HPF). All images were acquired as a ten slice Z-stack with scan speed of 800–1800 Hz and flattened as a maximum intensity projection prior to analysis. The wavelength of the laser to capture the signal for GFP and TMRE signals was 500–530 nm and 620–700 nm, respectively. Aggregates were counted per HPF by an observer blinded to condition.

### Statistics

Two-sided unpaired or paired, as appropriate, *t*-tests were used for statistical comparisons and all variances were of a similar magnitude except for death versus hypoxic exposure time curves (Fig. 3), where simultaneous curve-fitting was performed [48, 49]. Simultaneous curve-fitting forced the test RNAi death curve fit and the L4440 empty vector control curve fit to the same hypoxic exposure time producing 50% death (LT50) and *F*-tests were performed for statistical significance of the change in variance of the LT50 versus fitting each curve independently. Statistics were calculated using GraphPad Prism 6.01 (San Diego, CA, USA) for simultaneous curve-fitting, *t*-tests, or Excel 2007 for *t*-tests (Microsoft, Redmond, WA). Values are expressed as mean ± SD of at least three independent experiments. A minimum *p* value of ≤0.05 was considered significant and was Bonferroni-corrected for multiple comparisons where appropriate. Sample sizes for technical replicates for number of animals was based on the variability from animal to animal for fluorescence-based assays and was always ≥10 or a minimum of 30 animals, where a percent of the population was scored. Based on our previous experience with hypoxic death population assays biological replicates were at least three per condition, and if significant variability was observed among replicates more were performed until clear reproducibility was achieved. In terms of randomization, strains were not randomized as they are genetically identical and raised under environmentally identical conditions.

## Supplementary information

Table S1

Table S2

Figure S1

Figure S2

Supplementary Figure Legends

## Data Availability

The raw data from the screen of the RNAi strain set are available upon request. All other data generated and analyzed during this study are included in the published article. The unique *C. elegans* strains generated for this study are available upon request.
