# Supplementary material for: Effect of the mitochondrial unfolded protein response on hypoxic death and mitochondrial protein aggregation
Source: Cell Death Dis. 2021 Jul 15;12(7):711. doi: 10.1038/s41419-021-03979-z (PMC8282665; doi:10.1038/s41419-021-03979-z)
Supplement: Supplementary file 1 — Table S1 [file 41419_2021_3979_MOESM1_ESM.docx]

| **Supplementary Table S1. Five UPR^mt^-activating Hyp RNAis** | |  |  |
| --- | --- | --- | --- |
| **Gene name** | **Human ortholog*** | **Functional category** | **Subcellular localization^#^** |
| *opt-2/pept-1* | H+/oligopeptide symporter; human SLC15A1 (solute carrier family 15 member 1) and SLC15A2 (solute carrier family 15 member 2) | channel/transport | plasma membrane |
| *sucl-2* | SUCLG1 (succinate-CoA ligase alpha subunit) | metabolism | mitochondrion |
| *C33F10.12* | SLC25A3 (solute carrier family 25 member 3), ortholog to phosphate carrier protein | metabolism | mitochondrion |
| *pdha-1* | PDHA1 (pyruvate dehydrogenase E1 alpha 1 subunit) and PDHA2 | metabolism | mitochondrion |
| *R04F11.2* | ATP5ME (ATP synthase membrane subunit e) | metabolism | mitochondrion |
| *adapted from Wormbase (https://wormbase.org/) | |  |  |
| ^#^predicted by TargetP-2.0 Server (http://www.cbs.dtu.dk/services/TargetP/) or PredictProtein (https://predictprotein.org/) | | |  |
