# Supplementary material for: Effect of the mitochondrial unfolded protein response on hypoxic death and mitochondrial protein aggregation
Source: Cell Death Dis. 2021 Jul 15;12(7):711. doi: 10.1038/s41419-021-03979-z (PMC8282665; doi:10.1038/s41419-021-03979-z)
Supplement: Supplementary file 2 — Table S2 [file 41419_2021_3979_MOESM2_ESM.docx]

| **Supplementary Table S2. UPR^mt^-activating RNAis** | |  |  |
| --- | --- | --- | --- |
| **Gene name** | **Human ortholog*** | **Functional category** | **Subcellular localization^#^** |
| *letm-1* | LETM1 (leucine zipper and EF-hand containing transmembrane protein 1) | receptor/signaling | mitochondrion |
| *F15D3.6* | PRELID3A (PRELI domain containing 3A) and PRELID3B (PRELI domain containing 3B) | metabolism | cytoplasm |
| *pdhb-1* | PDHB (pyruvate dehydrogenase E1 subunit beta) | metabolism | mitochondrion |
| *mrpl-44* | MCCC2 (methylcrotonoyl-CoA carboxylase 2) | metabolism | mitochondrion |
| *wah-1* | AIFM1 (apoptosis inducing factor mitochondria associated 1) | cell death | mitochondrion |
| *dlst-1* | DLST (dihydrolipoamide S-succinyltransferase) | metabolism | mitochondrion |
| *tomm-22* | TOMM22 (translocase of outer mitochondrial membrane 22) | transport | mitochondrion |
| *dnj-21* | DNAJC19 (DnaJ heat shock protein family (Hsp40) member C19) | signaling | mitochondrion |
| *lpd-9* | Lipid storage | metabolism | cytoplasm |
| *cox-5B/cco-1* | COX5B (cytochrome c oxidase subunit 5B) | metabolism | mitochondrion |
| *tin-44* | TIMM44 (translocase of inner mitochondrial membrane 44) | transport | mitochondrion |
| *timm-17B.1* | TIMM17A (translocase of inner mitochondrial membrane 17A) and TIMM17B (translocase of inner mitochondrial membrane 17B) | transport | mitochondrion |
| *adapted from Wormbase (https://wormbase.org/) | |  |  |
| ^#^predicted by TargetP-2.0 Server (http://www.cbs.dtu.dk/services/TargetP/) or PredictProtein (https://predictprotein.org/) | | | |
