# Supplementary figures and images for: Effect of the mitochondrial unfolded protein response on hypoxic death and mitochondrial protein aggregation
Source: Cell Death Dis. 2021 Jul 15;12(7):711. doi: 10.1038/s41419-021-03979-z (PMC8282665; doi:10.1038/s41419-021-03979-z)

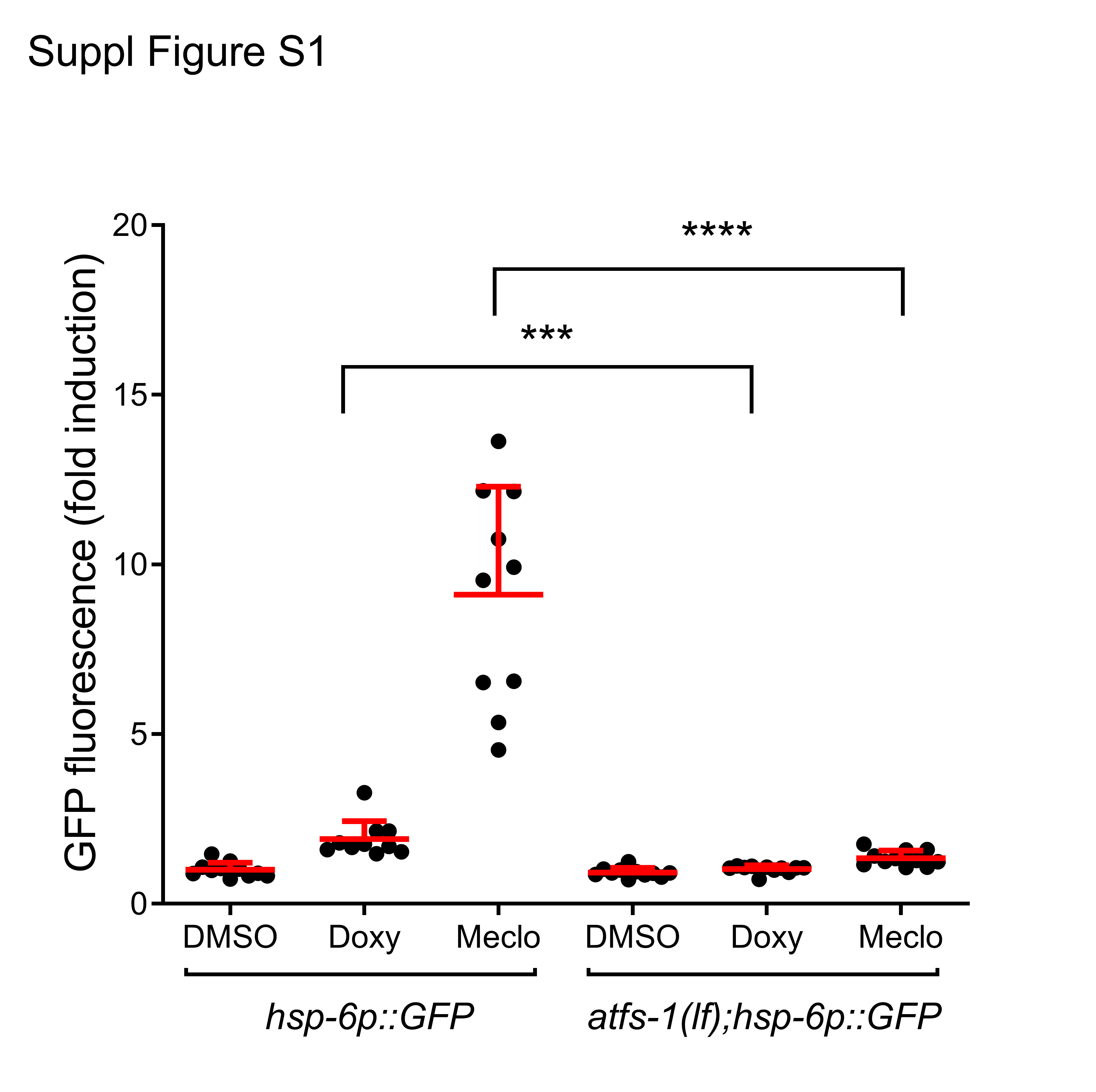

Supplement: Supplementary file 3 — Figure S1 [file 41419_2021_3979_MOESM3_ESM.tif]

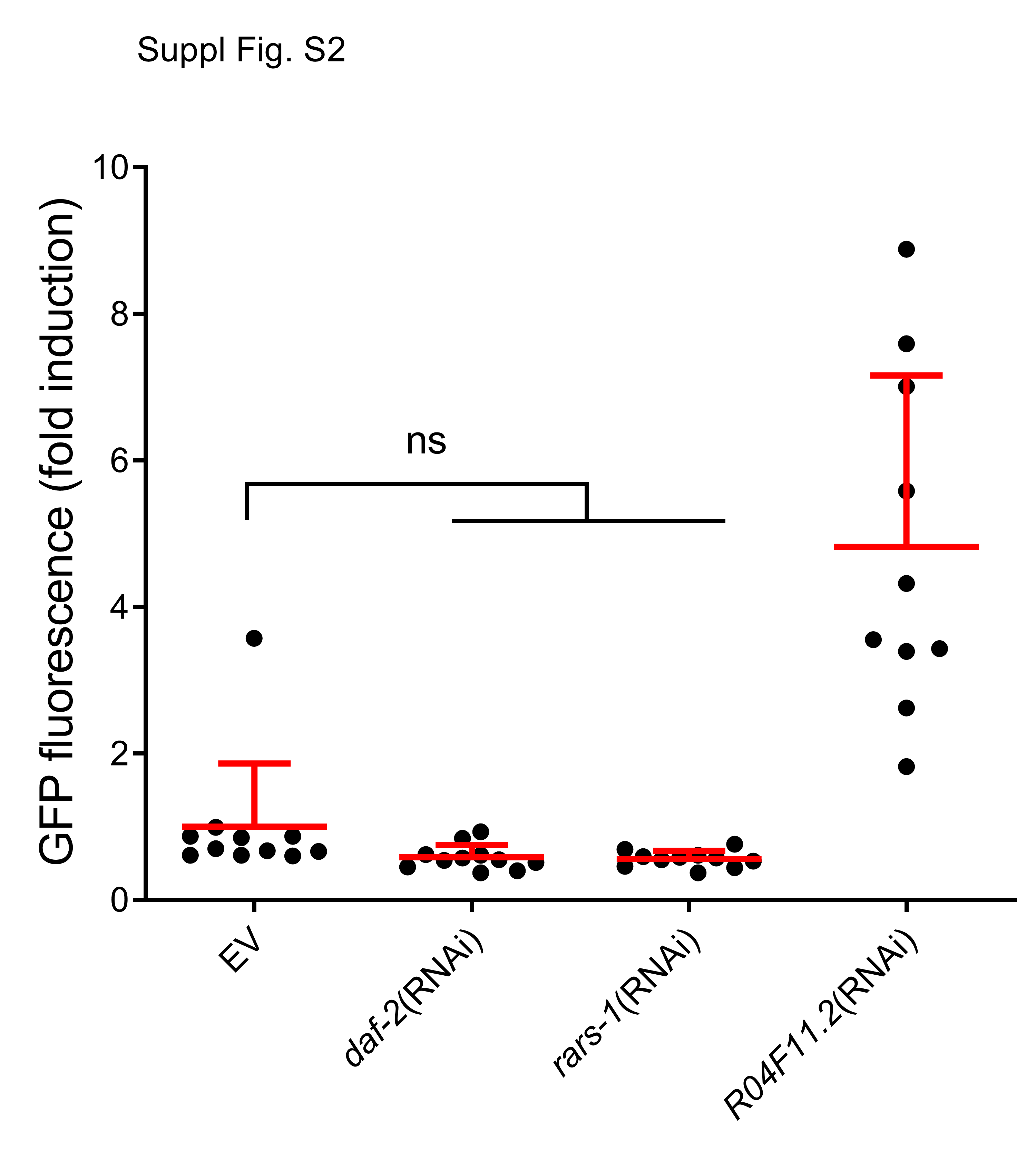

Supplement: Supplementary file 4 — Figure S2 [file 41419_2021_3979_MOESM4_ESM.tif]
