# Supplementary material for: Effect of the mitochondrial unfolded protein response on hypoxic death and mitochondrial protein aggregation
Source: Cell Death Dis. 2021 Jul 15;12(7):711. doi: 10.1038/s41419-021-03979-z (PMC8282665; doi:10.1038/s41419-021-03979-z)
Supplement: Supplementary file 5 — Supplementary Figure Legends [file 41419_2021_3979_MOESM5_ESM.docx]

**Supplementary Fig. S1: Activation of UPR^mt^ by doxycycline and meclocycline requires *atfs-1*.** Fold induction of *hsp-6p*::GFP fluorescence is the mean fluorescence relative to empty vector RNAi (L4440). (mean±SD, n=10 animals); ***, p<0.001, ****, p<0.0001, unpaired t-test.

**Supplementary Fig. S2: Knockdown of *daf-2* and *rars-1* do not activate UPR^mt^.** Fold induction of *hsp-6p*::GFP fluorescence is the mean fluorescence relative to empty vector RNAi (L4440) (n=10). R04F11.2 RNAi was used as a positive control for UPR^mt^ activation. (mean±SD, n=10 animals); ns – not significant at p<0.05, unpaired t-test.
